# Labor induction information leaflets—Do women receive evidence-based information about the benefits and harms of labor induction?

**DOI:** 10.3389/fgwh.2022.936770

**Published:** 2022-11-21

**Authors:** Peter von Dadelszen, Susan Tohill, Julie Wade, Jennifer A. Hutcheon, Janet Scott, Marcus Green, James G. Thornton, Laura A. Magee, the WILL Pilot Trial Study Group

**Affiliations:** ^1^Institute of Women and Children's Health, School of Life Course Sciences, Faculty of Life Sciences and Medicine, King's College London, London, United Kingdom; ^2^Department of Obstetrics and Gynaecology, University of British Columbia, Vancouver, BC, Canada; ^3^Maternity Services, Guy's and St Thomas' NHS Foundation Trust, London, United Kingdom; ^4^Sands, The Stillbirth and Neonatal Death Charity, London, United Kingdom; ^5^Action on Pre-eclampsia, Evesham, United Kingdom; ^6^Department of Obstetrics and Gynaecology, University of Nottingham, Nottingham, United Kingdom

**Keywords:** patient information, Morecombe Bay Report, Ockenden Report, evidence-to-implementation gap, labor induction

## Abstract

**Objectives:**

To determine the extent to which a sample of NHS labor induction leaflets reflects evidence on labor induction.

**Setting:**

Audit of labor induction patient information leaflets—local from WILL trial (When to Induce Labor to Limit risk in pregnancy hypertension) internal pilot sites or national-level available online.

**Methods:**

Descriptive analysis [*n* = 21 leaflets, 19 (one shared) in 20 WILL internal pilot sites and 2 NHS online] according to NHS “Protocol on the Production of Patient Information” criteria: general information (including indications), why and how induction is offered (including success and alternatives), and potential benefits and harms.

**Results:**

All leaflets described an induction indication. Most leaflets (*n* = 18) mentioned induction location and 16 the potential for delays due to delivery suite workloads and competing clinical priorities. While 19 leaflets discussed membrane sweeping (17 as an induction alternative), only 4 leaflets mentioned balloon catheter as another mechanical method. Induction success (onset of active labor) was presented by a minority of leaflets (*n* = 7, 33%), as “frequent” or in the “majority”, with “rare” or “occasional” failures. Benefits, harms and outcomes following induction were not compared with expectant care, but rather with spontaneous labor, such as for pain (*n* = 14, with nine stating more pain with induction). Potential benefits of induction were seldom described [*n* = 7; including avoiding stillbirth (*n* = 4)], but deemed to be likely. No leaflet stated vaginal birth was more likely following induction, but most stated Cesarean was not increased (*n* = 12); one leaflet stated that Cesarean risks were increased following induction. Women's satisfaction was rarely presented (*n* = 2).

**Conclusion:**

Information provided to pregnant women regarding labor induction could be improved to better reflect women's choice between induction and expectant care, and the evidence upon which treatment recommendations are based. A multiple stakeholder-involved and evidence-informed process to update guidance is required.

## Introduction

Induction of labor is increasingly common in the UK. According to national UK maternity statistics, inductions have increased from 20.4% of births in 2007–8, to 31.6% in 2017–18 (1). While nearly one third of pregnant women in the UK now have an induction of labor (1), other women are offered induction but decline. Evidence to date supports labor induction at term in terms of reducing rates of Cesarean birth and gestational age-dependent pregnancy complications (e.g., pre-eclampsia) (2, 3).

The harms and benefits of labor induction vs. expectant management are being evaluated in a number of National Institute for Health Research (NIHR)-funded trials, both ongoing (4, 5) and complete (6). One of these is the ongoing WILL Trial (When to Induce Labor to Limit risk in pregnancy hypertension) (ISRCTN77258279) that is recruiting and randomizing hypertensive pregnant women without pre-eclampsia to either planned delivery at 38^+0^ to 38^+3^ weeks (by labor induction or elective Cesarean) or expectant care until at least 40^+0^ weeks, unless delivery is indicated earlier by clinical need (5).

When discussing labor induction, women and families seeking NHS care are usually provided with local patient information about induction and local protocols for induction procedures in hard copy (leaflet) or online, through a hospital/Trust website. During the WILL internal pilot phase, discussions with individuals and with study site teams identified inter-site differences in labor induction terminology, advice and processes, with an apparent general bias against induction, as recently highlighted in the Morecombe Bay and Ockenden Reports (7, 8). As clear, accurate, unbiased, and consistent information is required by women and their families to make well-informed decisions about labor induction as an option, we undertook an audit of WILL internal pilot site labor induction information leaflets and those available online from the National Institute for Health and Care Excellence (NICE) and the National Health Service (NHS).

## Methods

The WILL internal pilot recruited women at 20 sites. The two lead co-ordinating research midwives (ST, JW) contacted these sites and investigated their patient-facing materials to gather all available patient information leaflets regarding induction of labor; within trusts, these were available in hard copy in clinics and/or online in the same format.

We evaluated these leaflets against criteria from the NHS Shetland Protocol on the Production of Patient Information (9). While from 2010, this guidance outlines criteria for patient leaflet content, of which we focused on the criteria of general information, treatment process, and harms and benefits. The other seven criteria in this Protocol were not evaluated in our review as they were considered to be related to information presentation or were too vague to evaluate objectively (i.e., clear aims, be balanced and unbiased, refer to uncertainties, list all sources of information, and support shared decision-making).

General information was date of issue, and relevance to the intended audience, interpreted here as indications for induction. Process of induction criteria were: a description of how induction would be undertaken (i.e., location, methods, and likelihood of success), “treatment choices” (including “taking a break” and expectant care as alternatives), and what would happen without induction. Harms and benefits included the criterion of the effects of treatment choice on overall quality of life.

The pamphlet criteria used are consistent with the contemporaneous and specific NICE CG70 *Inducing labor* guidelines (2008) that recommended (number 1.1.1.2) that all of the following should be explained to women being offered labor induction: “the reasons for induction being offered; the when, where and how induction could be carried out; the arrangements for support and pain relief; the alternative options if the woman chooses not to have induction of labor; the risks and benefits of induction of labor in specific circumstances and the proposed induction methods; and that induction may not be successful and what the woman's options would be” (10).

Data were abstracted independently by four researchers (ST, JW, PvD, and/or LM), and circulated to sites for their verification before analysis. In addition, national patient-facing guidance was sought online, searching Google for “induction of labor leaflet UK” as an example of how women would look for information (February 20, 2020).

At the time that the survey was performed (2019), the relevant NICE guidance on labor induction (CG70) was published in 2008, with an evidence update most recently performed in 2013 (10). As such, evidence against which the leaflet content was compared was taken from the 58 systematic reviews in the Cochrane Database (11), that covered methods of induction (*N* = 39), timing of birth (*N* = 12), Cesarean as an alternative to induction (*N* = 2), duration of induction (*N* = 1), and place of induction (*N* = 1). These were supplemented by systematic reviews on indications for induction (12) and women's views (13–15). Since the survey was performed, NICE has released new labor induction guidance (16).

No ethics committee approvals were sought as all leaflets were documents readily available in the public domain.

Analyses were descriptive, and presented as median and interquartile range (IQR) for continuous variables and *N* (%) for categorical.

## Results

The 20 WILL internal pilot sites varied in size and location. They covered 11/15 Clinical Research Network regions in England, with one site in Wales. Six sites (30%) were intermediate in size (3,000–4,999 deliveries per year) and 14 (70%) large or very large (with at least 5,000 deliveries per year). Most sites (18, 90%) were tertiary referral centers.

There were 21 labor induction pamphlets reviewed. All WILL pilot sites contributed their leaflets, although two sites shared a single leaflet. Two additional national leaflets were identified online: “Inducing labor—your pregnancy and baby guide” (2017) and “Choices when pregnancy reaches 41 weeks” (2016) (17).

### General information

Most leaflets had sufficient detail to enable version control (20, 95%), by giving a version or document number (*n* = 15, 71%), publication date (*n* = 15, 71%; ranging from April 2014 to June 2019), or a “review-by” date (*n* = 3, 14%). Leaflet length was an average of seven pages (range 2–18). Some leaflets (*n* = 7, 33%) described the frequency with which induction is undertaken in maternity care, specifying a narrow range of 20–30%.

**Figure 1:**
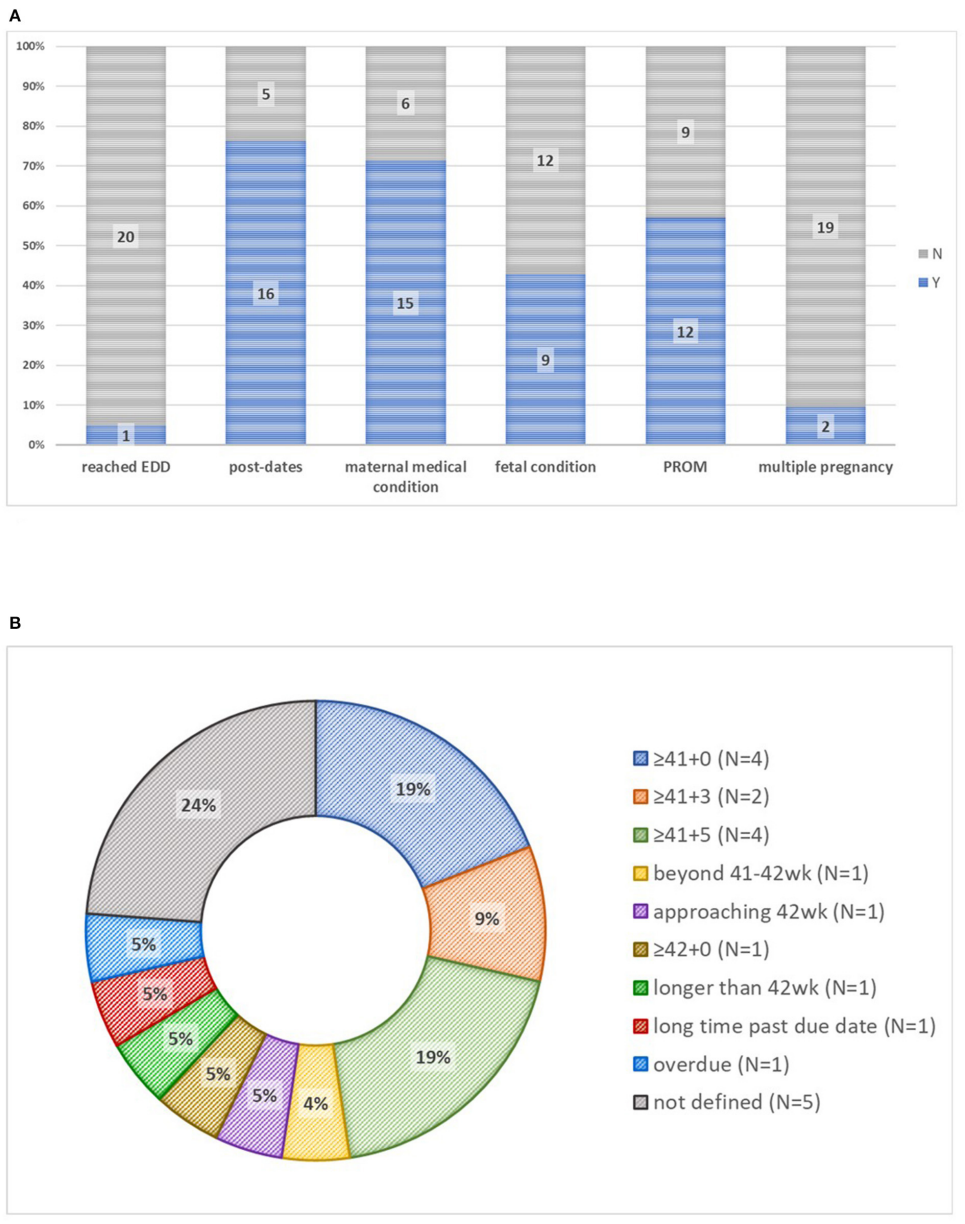
Indications for induction of labor and definitions of postdates in 21 patient information leaflets. **(A)** Indications for induction of labor. **(B)** Definitions of post-dates. EDD, estimated due date; PROM, prolonged rupture of membranes.

All leaflets described at least one indication for induction, most commonly post-dates gestational age (*n* = 16; 76%) and medical indications (*n* = 15, 71%) (Figure 1A). Definitions of post-dates varied widely (Figure 1B). Medical indications described were most often hypertension and diabetes (*N* = 11 each, 52%). The fetal indication for induction most frequently given was fetal growth restriction (*N* = 3, 14%). One leaflet gave maternal age of ≥40 years as an indication for induction at the expected date of delivery. One leaflet mentioned when induction is contraindicated, cited as non-cephalic fetal presentation or fetal growth restriction.

### Process of induction

Where women would be induced was almost always described (*n* = 18, 86%), in terms of delivery suite, triage unit, or induction unit. Women were usually informed about the potential for delays in starting the induction due to delivery suite workloads, limited staffing, and/or the needs of women with acute pregnancy complications (*n* = 16, 76%); one leaflet also mentioned consideration of limited neonatal intensive care unit capacity.

**Figure 2:**
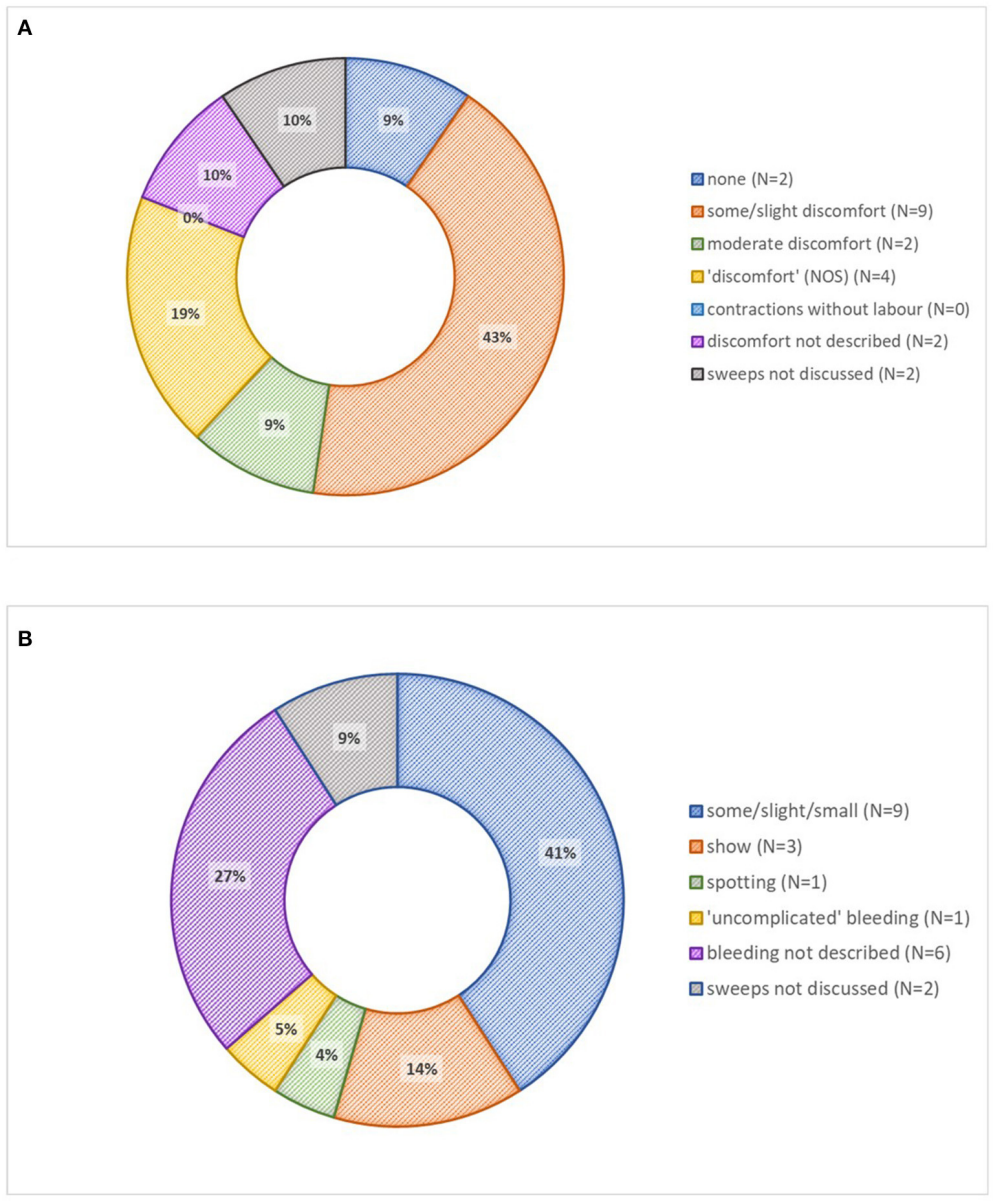
Membrane sweeping descriptions in 21 patient information leaflets. **(A)** Discomfort associated with membrane sweeping. **(B)** Bleeding associated with membrane sweeping. IOL, induction of labor; NOS, not otherwise specified.

Almost all leaflets discussed membrane sweeps (*n* = 19, 90%). One site had a separate information leaflet on the topic. How sweeps are conducted was usually described (*n* = 16, 76%), varying from an “internal examination” to a detailed account. Two leaflets considered sweeps to be an initial element of induction, but most (*n* = 17, 81%) explicitly described sweeps as an induction alternative that improves the likelihood of spontaneous labor onset. Fewer than half (*n* = 7, 33%) of leaflets presented the success rate of membrane sweeping, usually as “beneficial”, but one leaflet specified that 1 in 8 sweeps initiate labor. No leaflet described the likelihood of contractions without labor. Of 17 (81%) leaflets that discussed sweep-associated discomfort or pain, most (*n* = 9, 43%) rated it as “some” or slight” discomfort, two as moderate, and two as none (Figure 2A). Over half (*n* = 13, 62%) of leaflets stated that vaginal bleeding was possible, describing it as “show” or “some bleeding” (Figure 2B). If sweeping were unsuccessful, three leaflets (14%) mentioned the option for repeated sweeps.

**Figure 3:**
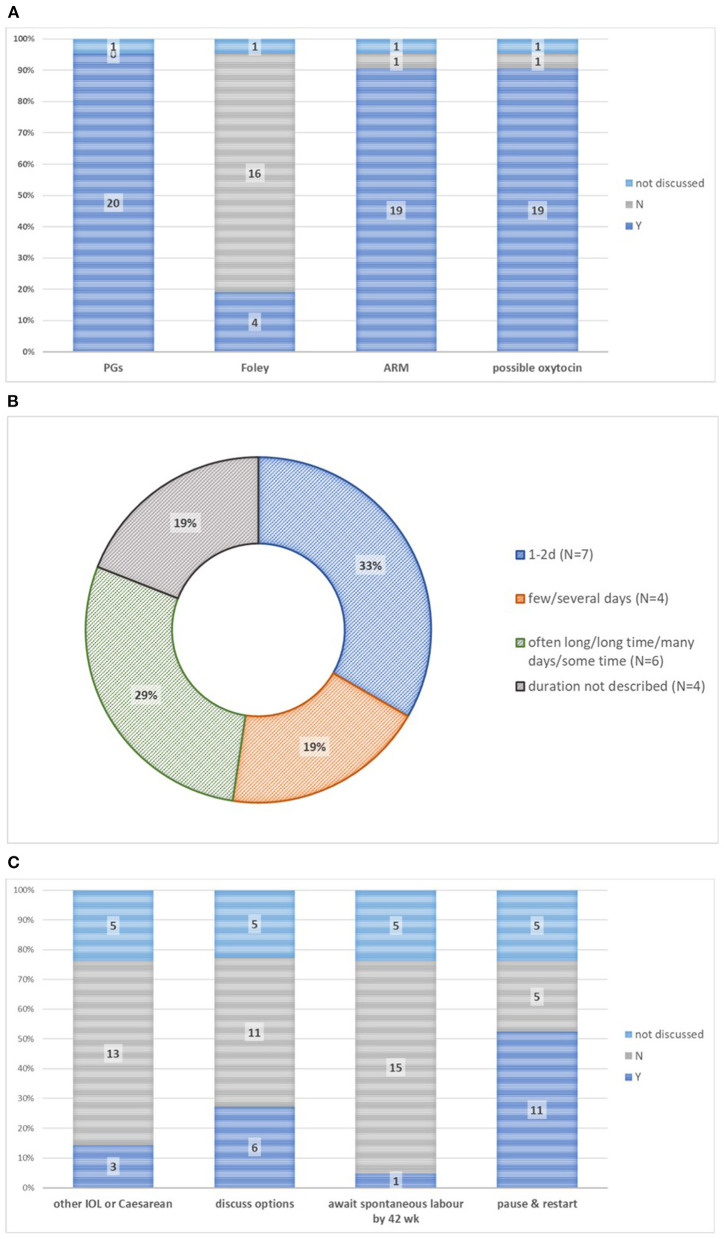
Process of induction in 21 patient information leaflets. **(A)** Methods of induction. **(B)** Time to onset of labor or birth. **(C)** Options if induction initially unsuccessful. ARM, artificial rupture of membranes; PGs, prostaglandin E_2_-based intervention; IOL, induction of labour.

Leaflets almost uniformly described the “how” of induction, with regards to use of a prostaglandin pessary, gel, or tablet (*n* = 20, 95%), balloon catheter (*n* = 4, 19%), or amniotomy (*n* = 19, 90%), with or without oxytocin (*n* = 19, 90%) (Figure 3A).

Few leaflets described adjuncts to labor induction [aromatherapy (*n* = 2, 10%) or hand-expressing (*n* = 1, 5%)].

The time from induction initiation to onset of active labor, with or without time to birth, was usually described (*n* = 17, 81%), but a few leaflets (*n* = 3, 14%) described only time to birth (Figure 3B). Time to active labor was variably described: as 1–2 days (*n* = 7, 33%), as “few/several” days (*n* = 4, 19%), or vaguely (*n* = 6, 29%; “often long”/“long time”/“many days”/“some time”) (Figure 3B). For comparison, few leaflets described early *spontaneous* labor, in terms of duration (“may take days”), pain (“irregular painful contractions”), or duration (“a long process with or without induced labor”) (*n* = 1, 5% each).

Induction success, defined as the onset of active labor, was presented by a minority of leaflets (*n* = 7, 33%), and described as “frequent” or in the “majority”, with “rare” or “occasional” failures.

In the case of failure to achieve active labor (Figure 3C), many leaflets (*n* = 11, 52%) described “taking a break” from the induction process, as an outpatient with maternal-fetal surveillance, should induction not be followed by onset of active labor within 24–48 h. Others described that if induction were not successful in initiating labor, options included Cesarean (*n* = 12).

No leaflet articulated that the alternative to induction, expectant care, may result in spontaneous labor, induction, or pre-labor Cesarean delivery. The alternative was usually presented as awaiting the onset of spontaneous labor (*n* = 13, 62%), during which time women were reassured that they would be offered ongoing surveillance (*n* = 12, 57%), described as “routine” or “intensive”. No pamphlet offered pre-labor Cesarean as an alternative to induction.

### Harms and benefits

No pamphlet used infographics to express harms or benefits. Only one pamphlet (5%) described benefits and harms in absolute terms, rather than as increased, decreased, or unchanged in incidence.

A minority of leaflets (*n* = 8, 38%) discussed potential benefits of induction, although these were often phrased as harms of *not* choosing induction. No leaflet stated that vaginal birth is more likely following labor induction compared with expectant care; one leaflet (5%) described unaltered vaginal delivery rates following induction compared with spontaneous labor. Few leaflets stated as a benefit an increased sense of control (*n* = 2, 10%) or a reduction in stillbirth risk (*n* = 6, 29%). Despite all leaflets presenting some indications for induction, fewer than half of leaflets presented an indication-specific benefit of induction, such as reduction in progression of maternal disease (*n* = 7, 33%).

**Figure 4:**
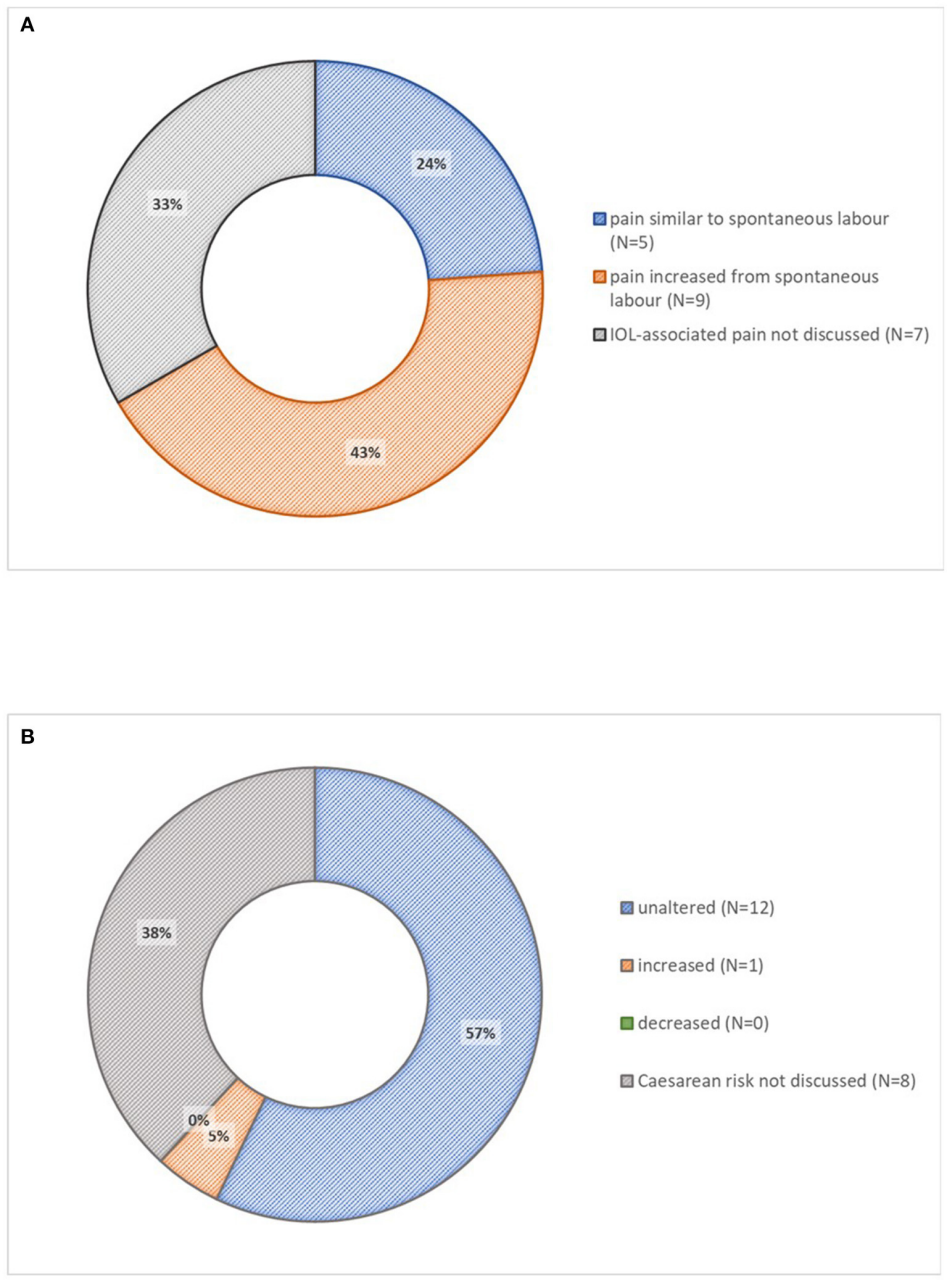
Described harms of induction of labor in 21 patient information leaflets. **(A)** Induction-associated pain. **(B)** Risk of Cesarean delivery. IOL, induction of labor.

Most leaflets discussed harms (*n* = 15, 71%); all discussed these harms in comparison with the alternative of spontaneous labor, not expectant care. Contraction intensity and frequency mentioned by most leaflets (*n* = 13, 62%) were usually described as mildly increased, with two leaflets describing the potential for an increase in fetal compromise leading to Cesarean. Pain was described as increased (*n* = 9, 43%) or similar (*n* = 5, 24%) (Figure 4A), but access to analgesia emphasized (*n* = 13, 62%). More vaginal exams (*n* = 1, 5%) and altered mobility (*n* = 5, 25%) were also mentioned. Most leaflets described Cesarean delivery risk as unaltered (*n* = 12, 57%); one leaflet described Cesarean risk as increased and none described it as reduced (Figure 4B). One leaflet described operative vaginal delivery risk as increased, although another described it as unaltered. Two leaflets described risks related to the method of induction specifically as vaginal irritation with vaginal prostaglandin (*n* = 2, 10%). Women's satisfaction with care was not usually mentioned (*n* = 2, 10%). Two leaflets (10%) mentioned that women may be less anxious if they have a date for induction. One leaflet stated that satisfaction is lower following labor induction, and the other presented reasons why satisfaction may be increased or decreased. Quality of life was not mentioned.

## Discussion

### Summary of findings

While most NHS leaflets reviewed discussed the why, how, and where of induction, and the potential for delays, some information was out-of-date (such as for mechanical methods of induction), some misleading (such as describing women's choice as being between labor induction and spontaneous labor), and other information often absent (such as benefits of induction). The procedure of membrane sweeping was usually presented as an alternative to induction, rather than as a mechanical method of induction. Benefits and harms were, with one exception, not expressed in absolute terms.

### Comparison with the literature

The key unanticipated finding was that induction leaflets compared labor induction with spontaneous labor, rather than with ongoing expectant care. This reflects the historical view taken in mode of delivery analyses, in which labor induction was compared with spontaneous labor. However, this approach was recognized to be methodologically biased as it led to a spurious association between labor induction and increased risk of Cesarean delivery, and failed to find an association between labor induction and a reduced risk of stillbirth (18–20). Systematic review comparing labor induction with expectant management (rather than spontaneous labor) has demonstrated that Cesarean birth is reduced by induction (14.9 vs. 17.0%; RR 0.88, 95% CI 0.84, 0.93; 157 trials and specifically at term, RR 0.87, 95% CI 0.82–0.92; 113 trials) (21), by induction at or beyond term in low-risk pregnancies, specifically (16.4 vs. 18.7%; RR 0.90, 95% CI 0.85, 0.95; 31 trials, 21,030 women) (2), and regardless of the indication or method of induction (21, 22). These findings have been supported by the ARRIVE trial of low-risk nulliparous women assigned to either labor induction at 39^+0^-39^+4^ weeks; induction was associated with fewer Cesarean births and cases of pregnancy hypertension (3). Importantly, women and care providers decide between induction and expectant care that may lead to either spontaneous labor onset, induction of labor, or semi-elective or emergency Cesarean, but none of the labor induction leaflets reviewed present women's choices in this way.

Given the wide range of definitions for “post-dates”, there appears to be uncertainty about how to interpret the Cochrane post-term induction reviews that were current at the time the leaflets were written (2, 23, 24). In the trials analyzed in this review, women were generally randomly assigned to *initiation of induction* at 41^+0^ (consequently *delivering* at 41^+3^ weeks) or provided with expectant management; this lag between initiation of induction and delivery has been observed consistently since the original post-term trial (2, 23, 24). Only four leaflets supported 41^+0^ as the optimal date for initiating a post-dates induction, with 10 offering an explicitly greater gestational age.

There is no evidence that “taking a break”, as described by half of leaflets, is either safe or effective. Of note, the risks of ongoing expectant care do not usually diminish with advancing gestational age (25). As adverse events thereafter cannot necessarily be predicted (26–28), “taking a break” may create medicolegal vulnerability.

Labor induction leaflets almost always discussed membrane sweeping as a way to avoid induction. While membrane sweeping is more easily provided and may be more cost-effective than using prostaglandins (29), it is intended to initiate labor (and, therefore, it is a form of induction) by causing a rise in circulating prostaglandin (that is similar to dinoprostone although of shorter duration) and requiring informed consent as advised by the Royal College of Midwives (30–34). While all induction leaflets were published prior to the 2019 Cochrane review of membrane sweeping (40 trials, 6,540 women), the evidence echoes the 2005 review that suggested caution about this procedure (35). The 2021 NICE inducing labor guideline now states that, “that membrane sweeping might make it more likely that labor will start without the need for additional pharmacological or mechanical methods of induction”, and advises maternity care providers to obtain verbal consent prior to undertaking the procedure (16). Women randomized to membrane sweeping (compared with no sweeping or a “sham” procedure treatment) were more likely to experience onset of labor (69.9 vs. 59.8%; RR 2.21, 95% CI 1.08, 1.34; 17 trials, 3,170 women) (29) and less likely to receive another form of induction (23.8 vs. 31.3%; RR 0.73, 95% CI 0.56, 0.94; 16 trials, 3,224 women) (29). However, these women were also more likely to experience an increase in contractions without labor (36.9 vs. 11.5%; RR 3.20, 95% CI 1.63, 6.28; 1 trial, 162 women) (36) and they were no more likely to achieve spontaneous vaginal birth (73.5 vs. 71.1%; aRR 1.03, 95% CI 0.99, 1.07; 26 trials, 4,538 women) (29). The potential benefits of membrane sweeping were no longer seen when low-quality trials or those with missing data were excluded. In another systematic review, membrane sweeping was not effective in achieving labor in women with prior Cesarean birth (2 trials, 361 women) (37). Furthermore, 20% of women who underwent membrane sweeps experienced pain beyond “discomfort” (2 trials, 320 women) (36, 38); this means that for every woman whose pregnancy is shortened by a few days, 4–6 women will experience discomfort and another 1–2 will experience pain (35).

The contemporaneous NICE guidance for labor induction was more than a decade old (2008). NICE recommended membrane sweeping as an “informal” method of induction prior to “formal” induction, and as a procedure that should be offered to nulliparous women at 40 weeks and all women at 41 weeks (to reduce the incidence of post-dates pregnancy), and every time a vaginal examination is undertaken to assess the cervix (39). The 2021 guidance now suggests discussing membrane sweeps with all women from 39^+0^ weeks, including the opportunity for repeated membrane sweeps (16). The latest surveillance report (January 2017) outlines the intention to update the guidance in a few specific areas, including mechanical methods of induction (under-represented in pamphlets) which offer advantages over pharmacological; Foley balloon catheter was associated with fewer Cesarean deliveries than dinoprostone insert (19.5 vs. 21.4%; RR 0.91, 95% CI 0.78, 1.07; 8 trials, 2,386 women) (40). However, the NICE CG70 update did not include membrane sweeping, information-sharing, or decision-making.

It was striking that the benefits of induction were described in fewer than half of leaflets. These are often condition-specific, such as a reduced risk of maternal progression of disease in hypertensive pregnancy. However, the risk of stillbirth increases with advancing gestational age even among low-risk nulliparous women, and among those at term and post-term gestational age, labor induction is associated with a reduced risk for stillbirth (0.2/1,000 vs. 1.7/1,000; RR 0.30, 95% CI 0.12, 0.75; 22 trials, 18,795 babies) and possibly fewer neonatal intensive care unit admissions (8.3 vs. 9.5%; RR 0.88, 95% CI 0.80, 0.96; 17 trials, 17,826 babies) (2). While the number-needed-to-induce to prevent one perinatal death is high (i.e., 544, 95% CI 441, 1,042), this conversation is important to have with women and with policy-makers, as the AFFIRM trial failed to demonstrate benefit from fetal surveillance by maternal monitoring of changes in fetal movements and intervention for reduced fetal movement (41).

The harms of induction were detailed. Induction is probably associated with more use of analgesia, but inconsistently, and pain scores were not reported in the recent Cochrane review. The recent ARRIVE trial of low-risk nulliparous women reported significantly lower pain scores associated with labor induction compared with expectant management (3).

While addressed by few pamphlets, women are not less satisfied when induced. In the term PROM trial, women who were induced (compared with women receiving expectant care) were less likely to report either that there was nothing they liked about their treatment or that the treatment caused additional worry, they were more willing to participate in the study again, and more often felt reassured (42). Other trials have found greater satisfaction associated with induction, or no difference compared with expectant care (43–45). To improve women's satisfaction, strategies such as decision aids, antenatal class redesign, and clinician communication training may be useful to address unconscious bias and improve the quality of information available to women and their capacity for informed decision-making (14).

In these authors' opinion, these findings, and the difficulty in seeing this manuscript published in a British journal, support the findings of the Morecombe Bay and Ockenden Reports (7, 8). Both reports criticized underlying philosophies of pursuing “normal childbirth ‘at any cost', ” despite countervailing evidence in support of timely surveillance and interventions with measured excesses in adverse maternal and perinatal outcomes.

### Strengths and weaknesses

Strengths of this analysis include comprehensive review of leaflets from sites active in timing of birth research, contributions from sites of varying sizes and locations, site confirmation of the accuracy of leaflet content from central abstraction, and public and patient involvement in review. Also, we benchmarked our information against high-quality and recent syntheses of trials conducted at term gestation, when women are most likely to be presented with the option of induction to prevent complications, rather than induction for clinical need.

Weaknesses include provision of leaflet content from 20 sites; while chosen to be representative of UK sites for an internal pilot trial, they do represent a minority of the more than 150 national maternity centers. We undertook no formal assessment of agreement, but double-checked agreement with sites for all site-level information before analysis. Our focus was on content, so we did not evaluate the suitability of the writing for patient education. We acknowledge that decisions are unlikely to be based on evidence-based patient information leaflets alone, although this is a starting point; women may use multiple sources of information, including online resources and friends and family, and clinicians may supplement information with face-to-face discussion, particularly for less common indications, site-specific resources (such as access to a birthing pool, home induction, or wireless telemetry) or to take into account women's views and preferences as part of shared decision-making. Finally, while we had two patient organization representatives and another patient representative to provide input into interpretation of the leaflet information, it was beyond the scope of this project to ask women themselves what they thought of the leaflets, what they would wish to see that was not available, factors that may influence their decisions, or the extent to which shared decision-making occurred, all important issues.

While not formally assessed in this study, the language used in the pamphlets was variably technical in nature. It is important that the UK and international maternity care community speaks with a common language with common definitions (e.g., post-dates and the labor-inducing intent of membrane sweeping) made accessible to women and their families as they engage in discussions around the initiation of giving birth.

## Conclusion

With the absence of an accurate predictive test for term stillbirth, and the recent AFFIRM trial finding that surveillance of and intervention for reduced fetal movements did not reduce stillbirth, there is a renewed focus on timed birth, initiated by labor induction, to reduce this devastating outcome for women and their families (2, 41). To make decisions that are right for them, women and their families must receive evidence-based, balanced information that weighs labor induction (or elective Cesarean) against ongoing expectant care with regards to potential benefits and harms, including pain, mode of delivery, complications, satisfaction with care, and personal views. There is no doubt that there are condition-specific indications for induction that require specific discussion of benefits and harms, and that there is a role for practical information once a decision to proceed with induction has been made; however, there is nevertheless a need for a generic national leaflet, based on current evidence and agreed by multidisciplinary stakeholders—most importantly, women, with a diverse range of backgrounds and experiences.

## Data availability statement

Publicly available datasets were analyzed in this study. This data can be found here https://www.birmingham.ac.uk/research/bctu/trials/womens/will/investigators/patient-info-leaflets.aspx.

## Author contributions

The study was designed and co-ordinated by LM, ST, JW, and PD. Data were collated by ST and JW. Analyzed by ST, JW, and PD. The first draft of the manuscript was written by LM and PD. Patient and public involvement and engagement was provided by JS and MG. All authors contributed to future revisions and the final submission version of the manuscript.

## Funding

This study was funded by the National Institute for Health Research (NIHR) Health Technology Assessment (HTA) Program (HTA ref: 16/167/123).

## Conflict of interest

Author MG was employed by Action on Pre-eclampsia. The remaining authors declare that the research was conducted in the absence of any commercial or financial relationships that could be construed as a potential conflict of interest.

## Publisher's note

All claims expressed in this article are solely those of the authors and do not necessarily represent those of their affiliated organizations, or those of the publisher, the editors and the reviewers. Any product that may be evaluated in this article, or claim that may be made by its manufacturer, is not guaranteed or endorsed by the publisher.

## Author disclaimer

The views expressed are those of the author(s) and not necessarily those of the NIHR or the Department of Health and Social Care.

## Appendix

**Table 1:** WILL Pilot Trial Study Group.

**From Clinical Investigator and Trial Management Groups**
Laura A. Magee (Chief Investigator), Peter von Dadelszen, Jon Dorling, Marcus Green, Jennifer Hutcheon, Ben W. Mol, Janet Scott, Joel Singer, Jim Thornton, Sue Tohill, Julie Wade
**From WILL trial pilot sites**
**Birmingham Women's Hospital**, Birmingham: Sophie-Anna Dann, Chloe O'Hara, Diane Whitehouse, Lesley Brittain, Phern Adams
**Bradford Teaching Hospitals NHS Foundation Trust**, Bradford: Sudeepthi Kakara, Jennifer Syson, Jenny Eedle, Kari Swettenham, Diane Farar
**Croydon University Hospital**, Croydon: Bini Ajay, Geraldine Upson
**The James Cook University Hospital**, Middlesbrough: Deepika Meneni, Hazel Alexander, Helen Harwood, Kerry Hebbron, Lynn Whitecross, Mary Hodgers, Shilpa Mahadasu
**University Hospitals of Leicester NHS Trust**, Leicester: Cornelia Wiesender, Beverley Cowlishaw, Claire Dodd, Gina Mulheron, Magdalena Kierzenkowska, Molly Patterson, Patricia Amos, Rupa Modi, Sharon Marie Bates, Sharon Raper, Manita Joshi, Andrea Goodlife
**Liverpool Women's Hospital**, Liverpool: Umber Agarwal, Ruth Cockerill, Amy Mahdi, Caroline Cunningham, Michelle Dower, Sian Andrews, Siobhan Holt
**Norfolk and Norwich Hospital NHS Foundation Trust:** Beth Gibson, Laura Harris
**North West Anglia NHS Foundation Trust**: Charleen Lia (PI, **Hinchingbrooke Hospital**, Hinchingbrooke), Coralie Huson, Jodi Carpenter
**Nottingham City Hospital**, Nottingham; Jim Thornton, Carys Smith, Harriet Anderson, Jane Cantliffe
**Princess Anne Hospital**, Southampton: Karen Brackley, Linden Stocker, Fiona Walbridge, Jane Forbes, Nicki Martin
**Queen's Medical Centre**, Nottingham: Jim Thornton, Catriona Hussain, Lesley Hodgen, Nia Jones
**Royal United Hospital**, Bath: Nisha Verasingam, Annette Moreton, Catherine Bressington, Jennifer Pullen, Mel Rich, Sara Burnard, Wendy Duberry
**Singleton Hospital**, Swansea: Madhuchanda Dey, Pauline Bird, Sharon Jones
**South Tyneside General Hospital & Sunderland Royal Hospital**, South Tyneside & Sunderland NHS Foundation Trust, Sunderland: Aarti Ullal, Ashleigh Price, Eileen Walton, Gemma Parish, Janet Scollen, Judith Ormonde, Kirsten Herdman, Lesley Hewitt, Lucy Rowland
**St James's University Hospital**, The Leeds Teaching Hospitals NHS Trust, Leeds: Jonathan Nelson, Carina Craig, Del Endersby, Jayne Wagstaff, Kate Robinson, Kathryn McNamara
**St Mary's Hospital, Manchester**: Jenny Myers, Meg Hyslop-Peart, Clare Waters
**St Mary's Maternity Unit, Poole Hospital**, University Hospitals Dorset NHS Foundation Trust: Latha Vinayakarao, Louise Melson, Stephanie Grigsby, Susara Blunden
**St Thomas' Hospital**, Guy's and St Thomas' NHS Foundation Trust, London: Alice Lewin, Declan Symington, Hayley Tarft, Jessamine Hunt, Julie Wade, Sue Tohill, Zoe Vowles
**West Middlesex University Hospital**, Chelsea and Westminster Hospital NHS Foundation Trust: Joanna Girling, Amy Barker
**York Hospital**, York: Jacqueline Tang, Kate Howard
